# Effect of tea catechins with caffeine on energy expenditure in middle-aged men and women: a randomized, double-blind, placebo-controlled, crossover trial

**DOI:** 10.1007/s00394-019-01976-9

**Published:** 2019-05-06

**Authors:** Shun Katada, Aya Yanagimoto, Yuji Matsui, Masanobu Hibi, Noriko Osaki, Shigeru Kobayashi, Yoshihisa Katsuragi

**Affiliations:** 1 Biological Science Research Laboratories, Kao Corporation, 2-1-3 Bunka, Sumida, Tokyo, 131-8501 Japan; 2 Department of Surgery, Tokyo Rinkai Hospital, 1-4-2 Rinkai-cho, Edogawa, Tokyo, 134-0086 Japan; 3 Health Care Food Research Laboratories, Kao Corporation, 2-1-3 Bunka, Sumida, Tokyo, 131-8501 Japan

**Keywords:** Body temperature, Energy expenditure, Middle-aged men and women, Resting metabolic rate, Tea catechins

## Abstract

**Purpose:**

It has been reported that tea catechins increase energy metabolism, but their effect on resting metabolic rate (RMR) remains under debate. This study aimed to examine the effect of repeated intake of tea catechins on energy metabolism in the resting state in middle-aged men and women.

**Methods:**

A total of 30 middle-aged men and women [13 women; age (mean ± SD) 52 ± 4 years; BMI 21.9 ± 2.2 kg/m^2^] were recruited. A randomized, double-blind, crossover study was conducted using a tea catechin-enriched beverage (611 mg catechins, 88 mg caffeine) and a placebo beverage (0 mg catechins, 81 mg caffeine) as test beverages. After 2 weeks of continuous test beverage intake, fasting RMR and energy expenditure (EE) after the ingestion of test beverage were measured. Measurements of forehead temperature (proxy for core temperature) and skin temperature were also obtained simultaneously.

**Results:**

Among participants who underwent measurements, 26 (10 women; mean age 52 ± 4 years; mean BMI 22.1 ± 2.1 kg/m^2^) were analyzed. The EE increased significantly after ingestion of the tea catechin beverage compared with the placebo beverage (placebo treatment: 5502 ± 757 kJ/day; catechin treatment: 5598 ± 800 kJ/day; *P* = 0.041). No between-treatment differences in fasting RMR or the respiratory quotient were detected. In addition, the forehead and skin temperature did not differ significantly between the placebo and catechin treatments.

**Conclusion:**

This study revealed that continuous intake of tea catechins with caffeine for 2 weeks significantly increased EE after ingestion of the tea catechin but not fasting RMR in middle-aged men and women.

**Clinical Trial Registry number and website:**

This trial was registered at www.umin.ac.jp/ctr/ as UMIN000025810 and UMIN000025811.

**Electronic supplementary material:**

The online version of this article (10.1007/s00394-019-01976-9) contains supplementary material, which is available to authorized users.

## Introduction

Obesity and overweight are major global health problems and critical risk factors for diabetes and cardiovascular disease [1]. Weight gain is attributed to excessive energy intake, as represented by overeating or decreased energy expenditure (EE), or both [2, 3]. An imbalance between energy intake and EE results in weight fluctuation, and a slightly positive energy balance per day (around 200 kJ/day) is thought to cause long-term weight gain [4].

Enhancement of EE by plant-derived ingredients, in addition to dietary restrictions and exercise, has attracted attention as a method for adjusting the energy balance [5]. Tea catechins (polyphenols contained in green tea) are one of these plant-derived ingredients with the potential to improve energy balance [6]. A synergistic effect of caffeine, which is also rich in green tea, was also reported for the thermogenic effect of catechin [7]. The intake of tea catechins with caffeine increases total energy expenditure (TEE) and fat utilization [8–10], and studies examining the effects of an acute single ingestion of tea catechins with caffeine report significant increases in EE compared with a placebo [9, 11], but other studies report inconsistent effects of continuous tea catechin with caffeine intake on fasting resting metabolic rate (RMR) [12, 13]. Moreover, although fasting RMR is known to decrease with age [14, 15], previous studies mostly targeted young healthy subjects to examine the effects of tea catechin with caffeine [8–11] and there are few studies describing the effects of tea catechin intake on fasting RMR and EE after ingestion in middle-aged individuals.

In this study, we examined the effects of continuous intake over 2 weeks of a tea catechin with caffeine on fasting RMR and EE after ingestion of the tea catechin in middle-aged men and women. We also measured the forehead and skin temperature simultaneously to confirm the effect of tea catechins with caffeine on body temperature.

## Methods

### Participants

Thirty healthy volunteers (17 men and 13 women; mean age 52 ± 4 years; mean BMI 21.9 ± 2.2 kg/m^2^) participated in this crossover trial. The inclusion criterion was 45–65 years of age. Exclusion criteria were as follows: treatment for severe disease; history or signs of a heart or cerebrovascular disease; taking therapeutic medicine for diabetes, hyperlipidemia or hypertension; smoker; heavy drinker; anemia; food allergies; and hypersensitivity to the test beverages. This trial was performed in accordance with the Declaration of Helsinki, and the trial plan was reviewed and approved by the local ethics committee (Kao Corporation, Tokyo, Japan). All subjects provided their informed, written consent to participate in the study. Recruitment of participants was conducted in January 2017. The treatment period was from February to April 2017.

### Test beverages

The test beverages were prepared by the Kao Corporation. The beverage referred to as the catechin beverage contained 611-mg catechins and 88-mg caffeine in 350 mL, and the caffeinated-placebo beverage contained 0-mg catechins and 81-mg caffeine in 350 mL (Table 1). The catechin beverage contained catechins comprising catechin (6%), epicatechin (5%), gallocatechin (22%), epigallocatechin (19%), catechin gallate (4%), epicatechin gallate (6%), gallocatechin gallate (18%), and epigallocatechin gallate (EGCg, 19%). The catechin beverage used in this study is commercially available in Japan, with the production standard of total tea catechins set to 174–190 mg/100 mL to guarantee a total tea catechin content of 540 mg/350 mL.

**Table 1:** Composition of the test beverages

	Placebo beverage	Catechin beverage
Catechin (mg)	0	34
Epicatechin (mg)	0	32
Gallocatechin (mg)	0	134
Epigallocatechin (mg)	0	118
Catechin gallate (mg)	0	25
Epicatechin gallate (mg)	0	39
Gallocatechin gallate (mg)	0	111
Epigallocatechin gallate (mg)	0	118
Total catechins (mg)	0	611
Caffeine (mg)	81	88

### Experimental design

This study was a two-phase, randomized, double-blind, placebo-controlled, crossover trial with a 2-week washout period between measurement periods (Supplemental Fig. 1). Subjects were randomized by stratified randomization to balance for BMI. The primary endpoint was energy metabolism after a 2-week intervention. After baseline measurements, subjects consumed one test beverage per day for 2 weeks while maintaining their usual lifestyle. Measurements on day 14 of phase 1 were obtained between 08:15 and 12:00. After a washout period of 2 weeks, in phase 2, subjects consumed the other test beverage per day for 2 weeks, again while maintaining their usual lifestyle. On day 14 of phase 2, measurements were obtained according to the same time-course used in phase 1. At each measurement period, energy metabolism and body temperature were measured before and after the test beverage ingestion and blood samples were obtained. During the trial, subjects were instructed to avoid intake of drinks/foods that contain a high amount of catechins, caffeine, and red pepper, and to maintain their usual exercise and dietary habits. Subjects also ate prescribed meals starting with breakfast up to dinner the day before each measurement period (10113 kJ/day; protein, 13E%; fat, 25E%, carbohydrates, 62E% for men and 8163 kJ/day; protein, 13E%; fat, 26E%, carbohydrates, 62E% for women, respectively) and were prohibited from performing strenuous exercise and drinking alcohol. The day before the measurement, subjects ate their prescribed meal by 20:00 and fasted thereafter.

### Anthropometrics and body composition

Body weight was measured using an automatic scale (TF-780, Tanita, Tokyo, Japan) and body composition was measured using whole-body dual-energy X-ray absorptiometry (DEXA, Hologic Inc., QRD 4500 W, Waltham, MA). Measurements of the fat-free mass (FFM), fat mass (FM), and fat ratio (%) were obtained using Hologic Systems Software according to the Hologic QRD 4500 User’s Guide.

### Indirect calorimetry

**Fig. 1:**
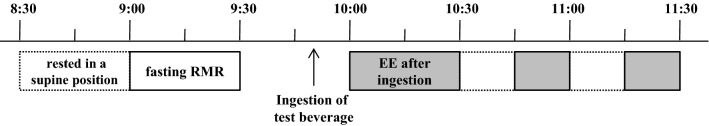
Diagrammatic representation of the study design

On measurement days, subjects were rested in a supine position on a bed, awake, in a fasting condition and in a room with a fixed environment (room temperature, 25 °C; humidity, 50%) for at least 30 min (8:30–9:00) before measurement of fasting RMR [16]. Using a hood-type respiratory gas analyzer (ARCO2000, Arco System, Inc., Chiba, Japan) [17], we assessed oxygen consumption ($\dot{V}$O_2_) and carbon dioxide production ($\dot{V}$CO_2_), and measured fasting RMR between 9:00 and 9:30 before the test beverage ingestion (Fig. 1). At 09:50, subjects consumed the test beverage within 5 min. Following this, EE after ingestion of the test beverage was measured three times at intervals of 15–30 min, between 10:00 and 11:30 as described above and the values from 10:00 to 11:30 were averaged to determine the EE after ingestion of the test beverage. $\dot{V}$O_2_ and $\dot{V}$CO_2_ were used to determine the EE [18] and respiratory quotient (RQ). The technical validity of the indirect calorimeter was repeatedly assessed by an alcohol combustion test throughout each trial on a weekly basis, repeated nine times. The coefficient of variation calculated from repeated measurement of the combustion rate was 1.2%.

### Measurements of body temperature and activity

In parallel with measurement of the EE, the forehead temperature (proxy for core temperature) was monitored by applying a temperature sensor (SpotOn™ Temperature Monitoring System, 3M, St Paul, USA) [19] to the forehead and using a data logger (LT-200, Gram, Saitama, Japan). The skin temperature was monitored using skin temperature sensors (LT-2N-12, Gram, Saitama, Japan) on the left subclavicular region and the left foot sole. A motion logger (Actigraph, MicroMini, AMI, NY, USA) was used to detect activity during the measurement of fasting RMR and EE after ingestion of the test beverage [20].

### Blood analysis

Blood was drawn at 9:30 am under fasting conditions. Fasting blood glucose and glycated hemoglobin (HbA1c) were measured using enzymatic methods. Fasting serum insulin, free triiodothyronine (T3), free thyroxine (T4), thyroid-stimulating hormone (TSH), progesterone, luteinizing hormone (LH), follicle-stimulating hormone (FSH) and estradiol were measured using a chemiluminescence immunoassay. Fasting serum total cholesterol, LDL cholesterol, HDL cholesterol, and triglycerides were measured using enzymatic methods. Fasting plasma noradrenaline was measured by HPLC and cortisol was measured by a chemiluminescence immunoassay. Measurements of parameters were performed by LSI Medience Corporation (Tokyo, Japan).

### Statistical analysis

Unless otherwise specified, all values are presented as mean ± SD. All cross-over data for the two treatments were compared using the paired t test (two-sided, *α* = 0.05). The relationship between FFM and fasting RMR was assessed using the Pearson correlation. Sample size was calculated based on a power analysis using preliminary data. A study group of 32 subjects was required for a power of 90% at a 5% significance level. All statistical analyses were performed with IBM SPSS Statistics for Windows, ver.19.0 (SPSS Inc., IL, USA).

## Results

Thirty healthy individuals participated in this study, and all participants completed the study. To accurately measure the fasting RMR and EE after ingestion of the test beverage, a motion logger Actigraph was used, and outliers in the dataset were detected on the basis of the interquartile range. Three subjects with an activity level greater than 1.5 × the interquartile range above the third quartile during the fasting RMR and EE after ingestion of the test beverage measurement were excluded from the analysis; one subject who took medicine during the measurement periods was also excluded and a per-protocol analysis was performed in twenty-six subjects (mean age 52 ± 4 years; mean BMI: 22.1 ± 2.1 kg/m^2^). The characteristics of the subjects are shown in Table 2. The rate of intake of the test beverage during the intervention period was 100% in both treatments.

**Table 2:** Subject characteristics at baseline

Age, y	52 ± 4
Sex, M/F, *n*	16/10
Height (cm)	166.4 ± 8.2
Body weight (kg)	61.6 ± 9.6
BMI (kg/m^2^)	22.1 ± 2.1
FFM (kg)	47.2 ± 8.4
FM (kg)	14.4 ± 4.3
Fat ratio (%)	23.4 ± 6.4
SBP (mmHg)	123 ± 19
DBP (mmHg)	76 ± 15
Glucose (mg/dL)	86 ± 7
HbA1c (%)	5.4 ± 0.2
Insulin (μU/mL)	2.8 ± 1.4
Total cholesterol (mg/dL)	197 ± 23
LDL cholesterol (mg/dL)	114 ± 25
HDL cholesterol (mg/dL)	60 ± 12
Triglycerides (mg/dL)	92 ± 41
Free T3 (pg/mL)	3.1 ± 0.3
Free T4 (ng/dL)	1.2 ± 0.2
TSH (μIU/mL)	1.140 ± 0.560
Noradrenaline (ng/mL)	0.33 ± 0.11
Cortisol (μg/dL)	7.8 ±2.5

Data are expressed as mean ± SD, *n* = 26

*DBP* diastolic blood pressure, *FFM* fat-free mass, *FM* fat mass, *HbA1c* glycated hemoglobin, *T3* triiodothyronine, *T4* thyroxine, *TSH* thyroid-stimulating hormone, *SBP* systolic blood pressure

### Energy metabolism

**Fig. 2:**
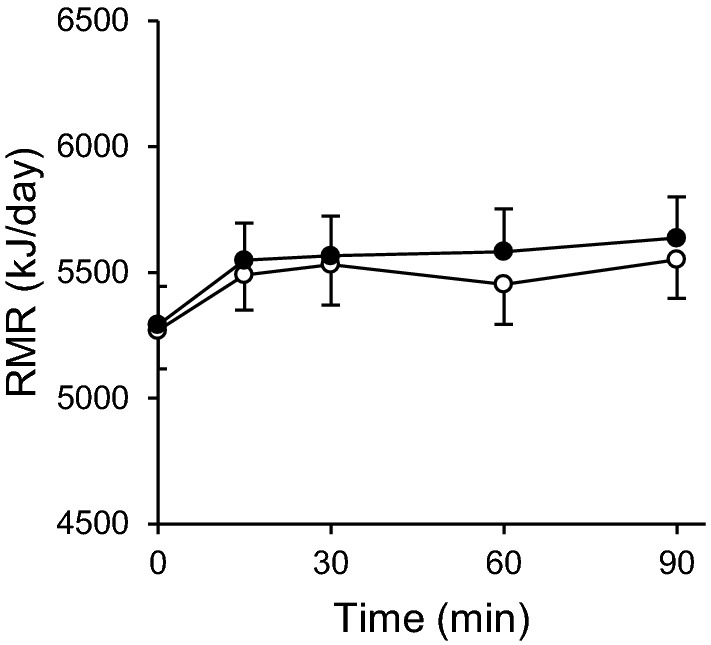
Time profile of fasting RMR and EE after ingestion following the 2-week intervention (placebo treatment: open circles; catechin treatment: closed circles). Data are expressed as mean ± SEM, *n* = 26, repeated measures ANOVA revealed significant treatment effect (treatment effect, *P* = 0.008; time effect, *P* < 0.001; treatment x time, *P* = 0.584)

A significant positive correlation was observed between the fasting RMR and the FFM of subjects in both the placebo treatment (*r* = 0.891, *P* < 0.001) and the catechin treatment (*r* = 0.856, *P* < 0.001). Table 3 shows fasting RMR, EE after ingestion of the test beverage and RQ following the 2-week intervention. Before ingestion of the test beverage, the fasting RMR did not differ significantly between the catechin treatment and the placebo treatment. On the other hand, the EE after ingestion of the test beverage was significantly higher in the catechin treatment than in the placebo treatment [placebo: 5502 ± 757 kJ/day; catechin: 5598 ± 800 kJ/day; *P* = 0.041; mean difference: 96 kJ/day (95% CI 4–188), Table 3 and Fig. 2]. A repeated-measures ANOVA revealed a significant treatment effect (*P* = 0.008), while there was no significant interaction between time and treatment (*P* = 0.584). Although incremental area under the curve (iAUC) at 45–90 min tend to be significantly higher in the catechin treatment (placebo: 8 ± 6 kJ/45 min; catechin: 11 ± 8 kJ/45 min; *P* = 0.099), no significant differences were observed at 0–90 min (placebo: 15 ± 10 kJ/90 min; catechin: 18 ± 13 kJ/90 min; *P* = 0.181, Supplemental Fig. 2). No significant differences were observed in RQ (Table 3).

**Table 3:** Resting metabolic rate (RMR), energy expenditure (EE) after ingestion of test beverages and respiratory quotient (RQ) after the 2-week intervention

	Placebo treatment	Catechin treatment	*P* value
Fasting RMR (kJ/day)	5269 ± 781	5291 ± 780	0.681
EE after ingestion (kJ/day)	5502 ± 757	5598 ± 800	0.041^a^
Changes in EE (kJ/day)	233 ± 209	307 ± 259	0.186
Fasting RQ	0.868 ± 0.041	0.874 ± 0.035	0.398
RQ after ingestion	0.854 ± 0.026	0.863 ± 0.035	0.104
Changes in RQ	− 0.015 ± 0.024	− 0.011 ± 0.030	0.525

Data are expressed as mean ± SD, *n* = 26

^a^Paired *t* test *P* < 0.05

### Body temperature

Table 4 shows changes in body temperature during energy metabolism measurements. Before ingestion of the test beverage, the forehead temperature and skin temperature did not differ significantly between the catechin treatment and the placebo treatment. Similarly, after ingestion of the test beverage, the forehead temperature and skin temperature did not differ significantly between the catechin treatment and the placebo treatment. Regression analyses showed no association between changes in RMR and changes in forehead temperature [placebo: *y* = 0.0001*x* + 0.117, *R*^2^ = 0.029, catechin: *y* = 0.0001*x* + 0.132, *R*^2^ = 0.063, *x*; changes in RMR (kJ/day), *y*; changes in forehead temperature (°C), *R*^2^; coefficient of determination].

**Table 4:** Forehead and skin temperature after the 2-week intervention

	Placebo treatment	Catechin treatment	*P* value
Forehead (proxy for core temperature)			
Fasting (°C)	36.66 ± 0.32	36.59 ± 0.29	0.114
After ingestion (°C)	36.80 ± 0.35	36.77 ± 0.34	0.384
Changes in temp (°C)	0.15 ± 0.16	0.17 ± 0.14	0.331
Left subclavicular skin			
Fasting (°C)	34.61 ± 0.56	34.63 ± 0.52	0.713
After ingestion (°C)	34.70 ± 0.56	34.70 ± 0.50	0.996
Changes in temp (°C)	0.09 ± 0.31	0.06 ± 0.32	0.693
Left foot sole skin			
Fasting (°C)	29.83 ± 2.27	29.94 ± 2.06	0.807
After ingestion (°C)	28.29 ± 1.50	28.23 ± 1.26	0.857
Changes in temp (°C)	− 1.54 ± 1.17	− 1.70 ± 1.20	0.388

Data are expressed as mean ± SD, *n* = 26

### Blood parameters and anthropometrics

Fasting blood parameters after the 2-week intervention are shown in Table 5. Glucose, insulin, total cholesterol, LDL cholesterol, HDL cholesterol, triglycerides, free T3, free T4, TSH, noradrenaline, and cortisol levels did not differ significantly between treatments. Although we could not confirm the menstruation cycles in female subjects, the progesterone, LH, FSH, and estradiol levels in the female subjects did not differ significantly between the treatments. Body weight and body fat were measured using an automatic scale after the 2-week intervention, and there are no differences between catechin and placebo treatment (body weight; placebo treatment: 61.4 ± 9.6 kg; catechin treatment: 61.5 ± 9.6 kg; *P* = 0.463, body fat; placebo treatment: 24.9 ± 5.6%; catechin treatment: 24.7 ± 5.6%; *P* = 0.289).

**Table 5:** Blood parameters after the 2-week intervention

	Placebo treatment	Catechin treatment	*P* value
Glucose (mg/dL)	86 ± 7	85 ± 7	0.256
Insulin (μU/mL)	3.3 ± 1.5	3.5 ± 1.7	0.552
Total cholesterol (mg/dL)	191 ± 23	191 ± 24	0.975
LDL cholesterol (mg/dL)	110 ± 23	110 ± 25	1.000
HDL cholesterol (mg/dL)	59 ± 13	59 ± 13	1.000
Triglycerides (mg/dL)	93 ± 39	96 ± 44	0.632
Free T3 (pg/mL)	3.2 ± 0.3	3.2 ± 0.3	0.658
Free T4 (ng/dL)	1.1 ± 0.2	1.1 ± 0.1	1.000
TSH (μIU/mL)	1.239 ± 0.730	1.249 ± 0.745	0.899
Noradrenaline (ng/mL)	0.32 ± 0.13	0.30 ± 0.09	0.206
Cortisol (μg/dL)	7.2 ± 1.9	7.1 ± 1.9	0.839
Progesterone (ng/mL)	4.9 ± 7.4	4.4 ± 6.8	0.819
LH (mIU/mL)	15.69 ± 16.36	16.47 ± 16.93	0.503
FSH (mIU/mL)	38.54 ± 38.94	40.89 ± 41.03	0.337
Estradiol (pg/mL)	61 ± 68	77 ± 94	0.387

Data are expressed as mean ± SD, *n* = 26 [progesterone, LH, FSH and estradiol were measured in female subjects (*n* = 10)]

*FSH* follicle-stimulating hormone, *LH* luteinizing hormone, *T3* triiodothyronine, *T4* thyroxine, *TSH* thyroid-stimulating hormone

## Discussion

The findings of the present study demonstrated that repeated intake of a tea catechin with caffeine for 2 weeks significantly increased EE after ingestion by 96 kJ/day (6 kJ/90 min) compared with the placebo beverage in middle-aged men and women. Our results suggest that repeated intake of tea catechins with caffeine over a 2-week increased the EE after ingestion of the catechin in the resting state by 1.7% in participants whose mean age was 52 years, which may have significant implications for obesity and weight gain associated with aging.

Previous studies examining the effects of tea catechin with caffeine intake on energy metabolism using a metabolic chamber reported increases in 24 h TEE after the beverage ingestion [8, 10]. On the other hand, conflicting findings are reported on the effects of tea catechins or EGCg with caffeine; in some studies, increases in the resting EE were observed in the tea catechin treatment compared with the control treatment [9, 11], whereas other studies detected no such differences [21, 22]. Although the present study revealed that continuous intake of a tea catechin-enriched beverage for 2 weeks significantly increased the EE after ingestion, further studies are needed to examine individual differences, changes in body composition due to continuous intake over a prolonged period of time, and the effects of dietary interventions.

In the present study, a 1.7% increase in the EE after ingestion was observed in the catechin treatment compared with the placebo treatment, which was statistically significant and consistent with previously reported thermogenic ingredients-induced increases in EE (2–4%) [5]. Similar to tea catechins, it has been reported that capsinoids also increase the EE and enhance the activation of brown adipose tissue [23, 24]. Galgani et al. observed an approximately 3% increase in the EE after pill ingestion in a parallel-group study using dihydrocapsiate [25]. Although these reported changes in the EE are relatively small, the resulting energy balance would be negative if there was no change in the dietary intake or exercise habits, thus having implications for long-term weight management.

Roughly, two-thirds of the fasting RMR is estimated to produce heat for homeothermy [26]. In the present study, we measured the non-invasive forehead (proxy for core) temperature as well as the skin temperature at the same time as the fasting RMR and EE after ingestion measurements were obtained; however, we detected no clear difference between the catechin and placebo treatments. In a previous study targeting Asian women with sensitivity to cold temperatures, fermented green tea suppressed decreases in limb temperature in mildly cold conditions (18–20 °C) [27]. Gosselin et al. examined the effects of EGCg with caffeine on thermogenesis under cold stimulation (15 °C), and reported that EGCg with caffeine increased nonshivering thermogenesis [28]. In the present study, energy metabolism measurements were obtained under 25 °C room temperature, which might make it difficult to detect a relatively small change in body temperature by tea catechin ingestion.

The test beverages using in this study contained approximately the same amount of caffeine (catechin beverage: 88-mg caffeine; placebo beverage: 81-mg caffeine), and it is likely that the EE after ingestion increase observed in this study is largely attributable to catechin itself. However, it is proposed that catechins and caffeine have a synergistic interaction on thermogenesis in brown adipose tissue [7, 29]. Thus, we cannot exclude a synergistic effect of tea catechins and caffeine to increase the EE after ingestion.

The mechanism of a tea catechin with caffeine-induced EE increase likely also involves the activation of AMP-activated protein kinase. It has been reported that tea catechins increase EE in mice through AMP-activated protein kinase α activation and enhancement of lipid metabolism [30]. In the present study, participants were in the fasting state for over 12 h at the time of test beverage intake, and because they were not given a meal or supplied with lipids and carbohydrates along with the test beverage, a decrease in the RQ might not have occurred. Moreover, tea catechins with caffeine reportedly activate brown adipose tissue in young people [9, 31]. Although it is possible that the tea catechin with caffeine-induced EE increase involves the activation of brown adipose tissue, further studies are needed to evaluate the effects of tea catechins with caffeine on brown adipose tissue in middle-aged individuals. Furthermore, the relatively short study period of 2 weeks might not have been sufficient to observe the effects of tea catechins with caffeine on body weight and body composition. To confirm these effects, a longer intervention period might be necessary.

In conclusion, the present study revealed that continuous intake of tea catechins with caffeine for 2 weeks significantly increased EE after ingestion but not fasting RMR in middle-aged men and women. Although a decline in energy metabolism due to aging may be inevitable, tea catechins with caffeine could help to maintain long-term energy metabolism, and hence, the management of body weight.

## Electronic supplementary material

Below is the link to the electronic supplementary material.

Supplementary material 1 (PDF 206 kb)

Supplementary material 2 (PDF 203 kb)

## Abbreviations

DBP: Diastolic blood pressure
DEXA: Dual-energy X-ray absorptiometry
EE: Energy expenditure
EGCg: Epigallocatechin gallate
FFM: Fat-free mass
FM: Fat mass
FSH: Follicle-stimulating hormone
HbA1c: Glycated hemoglobin
iAUC: Incremental area under the curve
LH: Luteinizing hormone
RMR: Resting metabolic rate
RQ: Respiratory quotient
SBP: Systolic blood pressure
T3: Triiodothyronine
T4: Thyroxine
TEE: Total energy expenditure
TSH: Thyroid-stimulating hormone
$$\dot{V}$$O_2_: Oxygen consumption
$\dot{V}$CO_2_: Carbon dioxide production
