# Supplementary figures and images for: Effect of tea catechins with caffeine on energy expenditure in middle-aged men and women: a randomized, double-blind, placebo-controlled, crossover trial
Source: Eur J Nutr. 2019 May 6;59(3):1163–70. doi: 10.1007/s00394-019-01976-9 (PMC7098939; doi:10.1007/s00394-019-01976-9)

## Flow Diagram

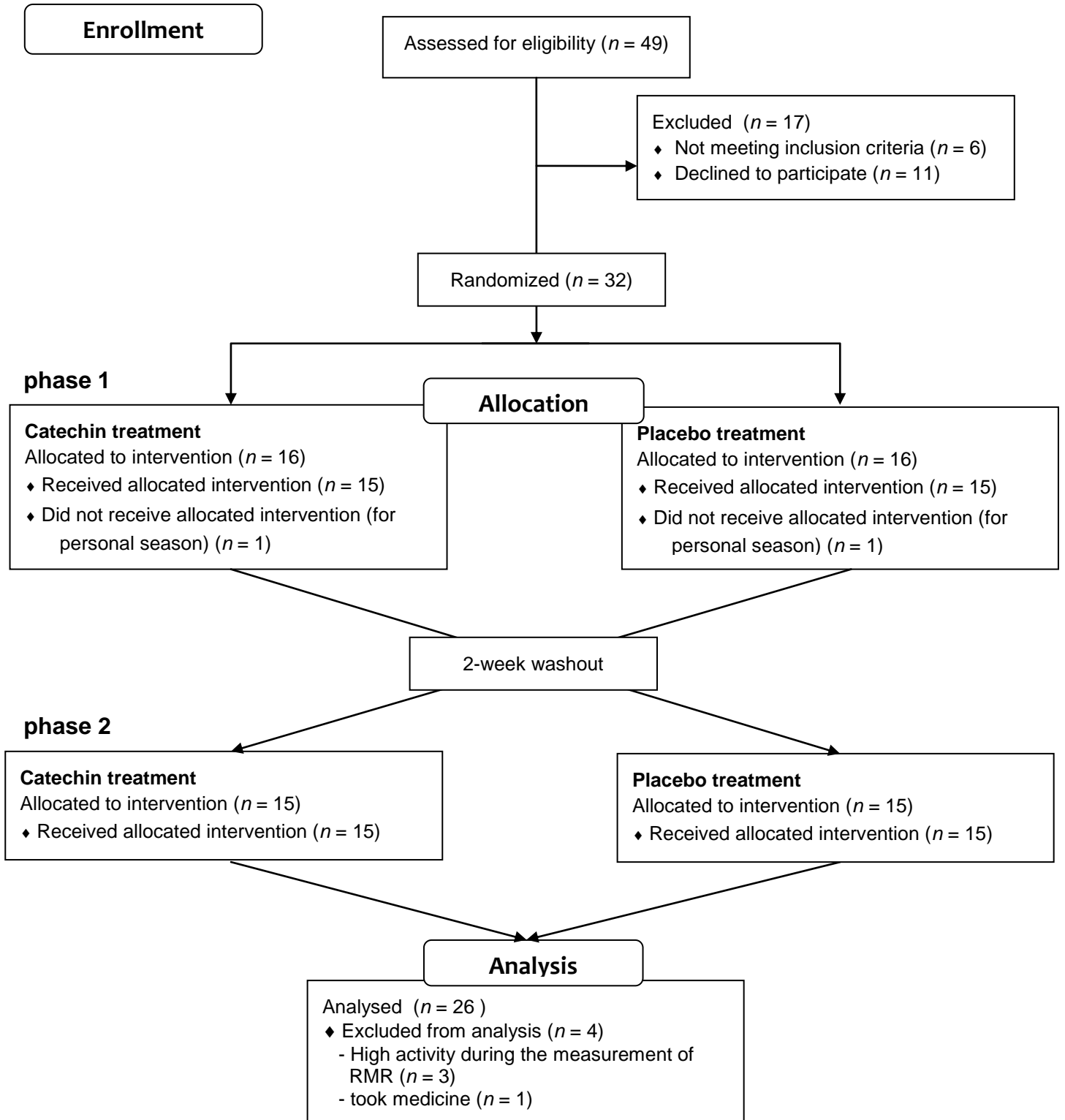

Supplement: Supplementary file 1 — Supplementary material 1 (PDF 206 kb) [file 394_2019_1976_MOESM1_ESM.pdf]
