# Supplementary material for: Effect of tea catechins with caffeine on energy expenditure in middle-aged men and women: a randomized, double-blind, placebo-controlled, crossover trial
Source: Eur J Nutr. 2019 May 6;59(3):1163–70. doi: 10.1007/s00394-019-01976-9 (PMC7098939; doi:10.1007/s00394-019-01976-9)
Supplement: Supplementary file 2 — Supplementary material 2 (PDF 203 kb) [file 394_2019_1976_MOESM2_ESM.pdf]

Supplemental Fig. 2

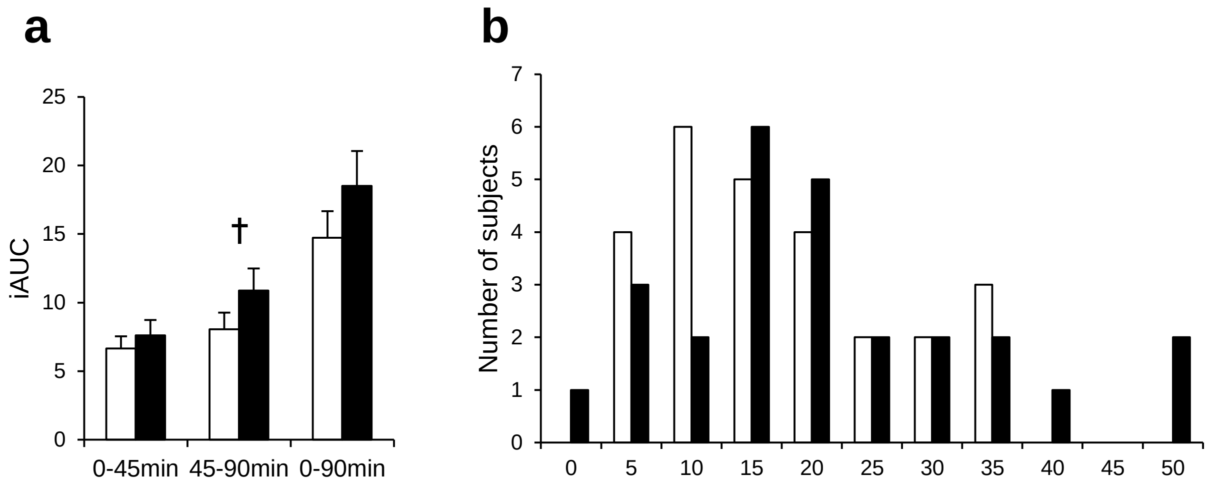

Supplemental Fig. 2
iAUC for energy expenditure following the 2-week intervention. **a** iAUC for energy expenditure at 0-45 min, 45-90 min and 0-90min (placebo treatment: white bar; catechin treatment: black bar). **b** shows histogram with a range of iAUC (kJ/90min) at 0-90 min (placebo treatment: white bar; catechin treatment: black bar). Data are expressed as means ± SEM, n = 26, † paired t-test $P < 0.1$.

Katada *et al.*
